# Draft Genome Sequences of Prototrophic and Biotin-Auxotrophic Fusarium langsethiae Strains Isolated from an Oat Grain in the Northern Region of Russia

**DOI:** 10.1128/mra.01250-21

**Published:** 2022-06-02

**Authors:** Aiko Tanaka, Daigo Takemoto, Ikuo Sato, Sotaro Chiba, Olga Gavrilova, Tatiana Gagkaeva

**Affiliations:** a Graduate School of Bioagricultural Sciences, Nagoya University, Chikusa, Nagoya, Japan; b All-Russian Institute of Plant Protection (VIZR), Pushkin, St. Petersburg, Russia; Vanderbilt University

## Abstract

Fusarium langsethiae is a suspected plant-pathogenic fungus causing cereal contamination with trichothecene mycotoxins. Here, we report the genome sequences of two *F. langsethiae* strains, MFG217701 (a prototroph) and MFG217702 (a biotin auxotroph), isolated from a grain of oat harvested in Russia.

## ANNOUNCEMENT

Fusarium langsethiae (1) is a fungus that asymptomatically infects a range of grain cereals, such as oat, wheat, and barley (2). There has been increasing interest in *F. langsethiae* because this species produces mycotoxins (T-2 and HT-2) which are type A trichothecenes (2–5). Previously, we detected a biotin auxotrophy in *F. langsethiae* strains collected from geographically different areas of Europe (6). Some *F. langsethiae* strains showed a disruption of normal growth, forming a poor, extremely sparsely branched colony in a synthetic medium unless biotin was added (6), indicating that biotin is available for *F. langsethiae* in colonized plant tissue. Auxotrophic strains are common in nature; thus, genetic changes in the basic metabolism of pathogens may give rise to changes in plant-fungi interactions, highlighting the importance of further investigations into the genomic changes and pathogenicity of auxotrophic strains.

**FIG 1:**
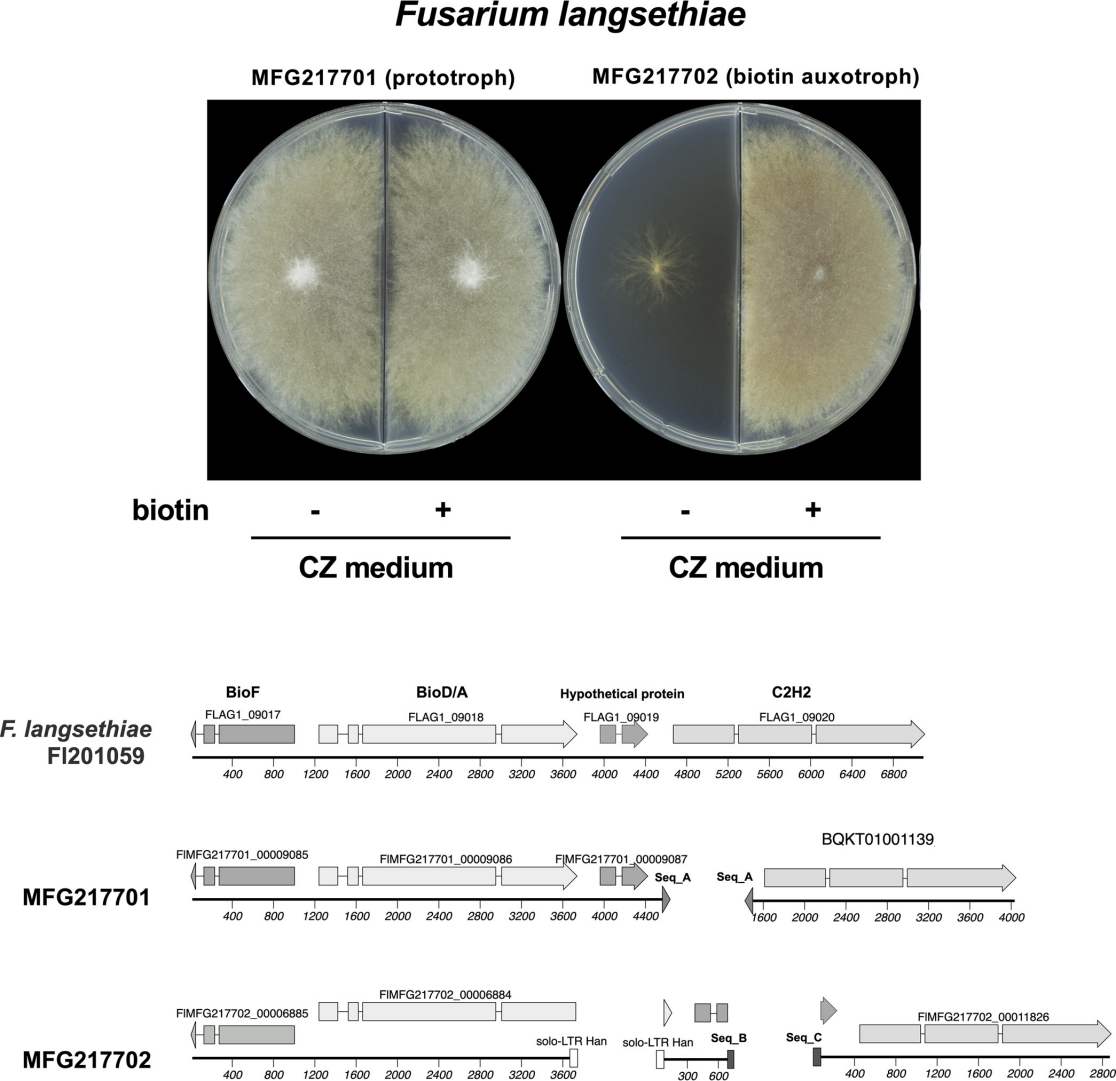
(Top) Growth of Fusarium langsethiae strains MFG217701 (prototroph) and MFG217702 (biotin auxotroph) on Czapek (CZ) medium or CZ medium supplemented with 0.01 mg/L biotin. The fungal strains were grown for 10 days at 24°C in the dark. (Bottom) Predicted biotin cluster in Fusarium langsethiae strains Fl201059, MFG217701, and MFG217702. The repeat sequences (solo-LTR Han, Seq_A, Seq_B, and Seq_C) disrupted the two open reading frames (ORFs) in the biotin-auxotrophic strain MFG217702. The ORFs in contig 1139 (GenBank accession no. BQKT01001139) were manually predicted. One of the repetitive sequences in the cluster is solo-LTR Han (13), and 65 and 63 copies of it were found in MFG217701 and MFG217702, respectively.

Here, we report the draft genome sequences of two *F. langsethiae* strains, MFG217701 (prototrophic) and MFG217702 (biotin auxotrophic), isolated from a sample of a single grain of oat harvested in northwestern Russia (Leningrad Oblast) in 2014. The Fusarium strains were single-spored and stored in the All-Russian Plant Protection Institute collection (St. Petersburg, Russia). Strain MFG217702 grows poorly on synthetic Czapek (CZ) medium; however, it exhibits colony phenotypes comparable with the prototrophic strain MFG217701 on CZ medium supplemented with biotin (Fig. 1).

Strains MFG217701 and MFG217702 were grown in potato sucrose broth, and genomic DNA was extracted using a DNeasy plant minikit (Qiagen, Germany). Whole-genome sequencing libraries were prepared using the MGIEasy FS DNA library prep set and sequenced on a DNBSEQ-G400RS instrument (MGI, China) using a whole-genome shotgun strategy. After quality filtering and trimming using fastp v0.20.0 with the options for removing adaptors and low-quality reads (7), paired-end data sets were assembled *de novo* using SPAdes v3.15.3 with the –careful option (8). The assembly quality was assessed using QUAST v4.4 (9) (Table 1). Default parameters were used except where otherwise noted.

**TABLE 1:** Summary of Fusarium langsethiae genome assemblies and annotations

Strain	Phenotype	No. of filtered paired reads	*N*_50_ (bp)	GC content (%)	Total no. of bases	No. of contigs	Sequencing coverage (×)	No. of genes	BUSCO analysis results^*a*^			
									Complete BUSCOs (%)	No. of BUSCOs	No. of missing BUSCOs	No. of fragmented BUSCOs
MFG 217701	Prototroph	45,369,500	76,622	48.2	38,427,502	3,556	351	12,467	99.0	4,447	32	15
MFG 217702	Biotin auxotroph	40,119,319	78,906	48.3	38,261,259	3,809	313	12,370	98.9	4,443	36	15

a Analysis conducted using the data set hypocreales_odb10.

A summary of the genome assemblies and annotations is shown in Table 1. Prediction of the protein-encoding genes was performed using BRAKER2 (10) with the annotated protein sequences of *F. langsethiae* strain Fl201059 as the input for model hints. The quality and completeness of the assembled genome were estimated using Benchmarking Universal Single-Copy Orthologs (BUSCO) with the data set hypocreales_odb10 (11). The gene cluster for biotin biosynthesis in *F. langsethiae* strain Fl201059 (12) containing BioF, BioD/A, a hypothetical protein, and C2H2 transcription factor genes were found in both genomes, although the gene cluster in the biotin-auxotrophic genome was interrupted by repetitive sequences that cause disruption of the genes encoding BioD/A and the hypothetical protein (Fig. 1). The gene cluster for trichothecene biosynthesis containing the Tri3 to Tri14 genes was found in both *F. langsethiae* genomes (MFG217701_contig0169 and MFG217702_contig0164).

### Data availability.

The *F. langsethiae* genome sequences were deposited at DDBJ/EMBL/GenBank under accession no. BQKT01000001 to BQKT01003556 (MFG217701) and BQKU01000001 to BQKU01003809 (MFG217702). The raw sequencing reads have been submitted to the DDBJ Sequence Read Archive (DRA) under accession no. DRR333357 (MFG217701) and DRR333358 (MFG217702).
